# The Latest Manifesto on Alcohol

**Published:** 1907-04-06

**Authors:** 


SURGEON GENERAL'S OFFICE

AUG. 8 -1907

The Hospital

A Journal of

The Medical Sciences and Hospital Administration.

New Series. No. 1, Vol. 1. No. 1073. Vol. XLII. SATURDAY, APRIL 6, 1907.

### The Latest Manifesto on Alcohol.

The question of the dietetic and medicinal position of alcohol is with us once again. A number of well-known physicians and surgeons, together with certain professors of physiology, have felt it their duty to issue a collective expression of opinion in favour of the value of alcoholic beverages both in health and disease. It is not difficult to identify the circumstances which have led to this step. Not many months have elapsed since a prominent metropolitan surgeon took the opportunity offered by the meeting of the British Medical Association at Toronto to denounce the use of alcohol in every shape and form; to proclaim its utter uselessness, and even its pernicious action, in the treatment of disease; and generally to condemn it as a dangerous and poisonous agent. Had these propositions been presented merely as the personal opinions of the orator, they might easily have been passed over as having no other attraction than a convenient re-statement of what the same authority had said on many previous occasions. But with them was associated at least a suggestion that such views represented the general attitude of the profession in this country, and the official position of the speaker lent, as was evident from the criticisms of the lay Press, an added significance to this statement. Considering what is the common custom and habit of medical practitioners on this side of the Atlantic in regard to the use of alcohol, at least in the treatment of disease, it is not a matter for wonder that a number of clinical teachers should, even somewhat late in the day, have determined to put on record views which are in direct conflict with those presented to the Toronto meeting. Now that the issue is joined, it is to be hoped that its discussion will be conducted in a fair and scientific fashion, and that from it will result both light and leading.

Turning to the manifesto itself, there can be no question of the professional weight and authority of the names attached to it. Yet about its genesis there is something mysterious. No indication is given of its origin, nor is there anything to suggest the method or principle on which the signatories have been selected. Further, it must be admitted, that though "fit," the signatories are certainly "few." The grand total numbers only sixteen, and even recognising the great authority of many of the individual names, such a total seems scarcely adequate to represent "the opinions of the leading clinical teachers," not to speak of "the great majority of medical practitioners." For the memorialists are not content to record their own views and to leave them to stand for what they may be worth. They must needs proclaim themselves as the voice alike of "the majority of" medical teachers and practitioners. "Codlin is the friend," they tell us, not the Toronto "Short." In these circumstances it is impossible to avoid inquiring why the names attached to the manifesto number only sixteen. "Leading" clinical teachers form a numerous constituency, and it would be interesting, and we think even important, to know how many hospital physicians and surgeons engaged in teaching were asked to sign the manifesto, and why it is that the published supporters are so few. Had the signatories confined themselves to a mere statement of opinion on the value of alcohol, no such inquiries would have been pertinent. But when they claim, as they do here, to represent the views of the profession, we are entitled to ask what steps they have taken to ascertain these views, and why it is that so many names, not less authoritative than their own, are not included in the manifesto they have issued. For such inquiries the responsibility lies with those who have gratuitously gone out of their way to announce themselves as self-proclaimed representatives of professional opinion.

The need for pressing a series of questions in regard to the origin and organisation of this manifesto is emphasised by the fact that it deals not only with the medicinal, but also with the dietetic value of alcohol. It will therefore inevitably be quoted in relation to the economic and ethical aspects of the temperance controversy. And we venture to say that the statement that "the moderate use of alcoholic beverages [as an article of diet] is, for adults, usually beneficial" ought not to be publicly announced as in harmony with the views of the leading clinical teachers of medicine and surgery in this country except upon the authority of an overwhelming weight of numbers. The members of the teaching staffs of our medical schools are well known and can be readily approached. Have they

W1
H069X

been approached? If so, how is the absence of their names to be reconciled with the claim of the manifesto to speak on their behalf? If these teachers have not been approached, what right have the signatories to speak for them? Until these questions are answered, the moral weight of the manifesto is nothing more than that of the individual opinions of those whose names it bears.

